# Use of a Combined SpO_2_/PtcCO_2_ Sensor in the Delivery Room

**DOI:** 10.3390/s120810980

**Published:** 2012-08-08

**Authors:** Serena Antonia Rubortone, Maria Pia De Carolis, Serafina Lacerenza, Iliana Bersani, Federica Occhipinti, Costantino Romagnoli

**Affiliations:** Division of Neonatology, Department of Paediatrics, Catholic University of Sacred Heart, Largo Gemelli 8, I-00168 Rome, Italy; E-Mails: sererubor@hotmail.it (S.A.R.); saralac@inwind.it (S.L.); ilianabersani@gmail.com (I.B.); federicaocchipinti@hotmail.it (F.O.); cromagnoli@rm.unicatt.it (C.R.)

**Keywords:** oxygen saturation, partial pressure of carbon dioxide, pulse oximeter, delivery room, TOSCA sensor, neonate

## Abstract

Arterial oxygen saturation (SaO_2_) and partial arterial pressure of carbon dioxide (PaCO_2_) are important respiratory parameters in critically ill neonates. A sensor combining a pulse oximeter with the Stow-Severinghaus electrode, required for the measurement of peripheral oxygen saturation (SpO_2_) and transcutaneous partial pressure of carbon dioxide (PtcCO_2_), respectively, has been recently used in neonatal clinical practice (TOSCA^500Ò^Radiometer). We evaluated TOSCA usability and reliability in the delivery room (DR), throughout three different periods, on term, late-preterm, and preterm neonates. During the first period (***period A***), 30 healthy term neonates were simultaneously monitored with both TOSCA and a MASIMO pulse oximeter. During the second period (***period B***), 10 healthy late-preterm neonates were monitored with both TOSCA and a transcutaneous device measuring PtcCO_2_ (TINA^Ò^ TCM3, Radiometer). During the third period (***period C***), 15 preterm neonates were monitored with TOSCA and MASIMO after birth, during stabilization, and during transport to the neonatal intensive care unit (NICU). Blood gas analyses were performed to compare transcutaneous and blood gas values. TOSCA resulted easily and safely usable in the DR, allowing reliable noninvasive SaO_2_ estimation. Since PtcCO_2_ measurements with TOSCA required at least 10 min to be stable and reliable, this parameter was not useful during the early resuscitation immediately after birth. Moreover, PtcCO_2_ levels were less precise if compared to the conventional transcutaneous monitoring. However, PtcCO_2_ measurement by TOSCA was useful as trend-monitoring after stabilization and during transport to NICU.

## 1. Introduction

Arterial oxygen saturation (SaO_2_) and partial arterial pressure of carbon dioxide (PaCO_2_) are two of the most important respiratory parameters in the evaluation of critically ill neonates. SaO_2_ is commonly estimated by pulse oximetry (SpO_2_), a non invasive technique widely used in neonatal intensive care units. Partial pressure of carbon dioxide (PCO_2_) can be assessed by sampling blood (PCO_2BGA_) or by noninvasive techniques such as transcutaneous monitoring (PtcCO_2_), which reduces the need for frequent blood drawing and painful punctures and provides a continuous estimation of PCO_2_.

**Figure 1.**
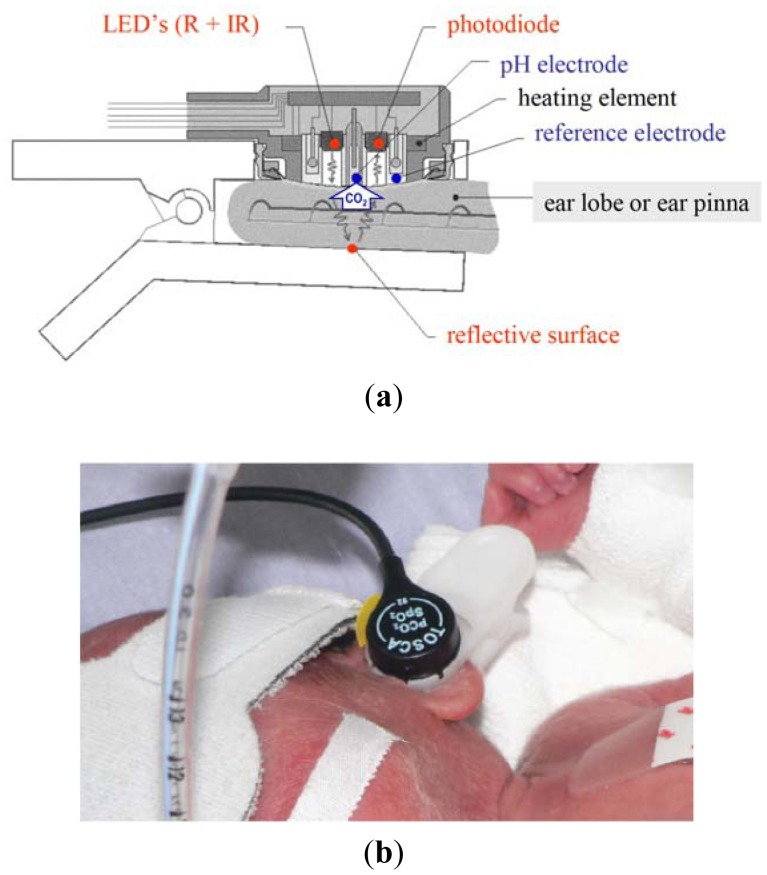
(**a**) TOSCA sensor combines an optical sensor for the measurement of SpO_2_ (coloured in red) with a Stow-Severinghaus type CO_2_ sensor (coloured in blue), and is also equipped with a heating element (coloured in black); image from Eberhard P. [2]. (**b**) Application of the TOSCA sensor at the ear pinna; image from Lacerenza S. [10].

As already described elsewhere [1], the TOSCA monitor combines a pulse oximeter of new technology (Masimo SET) with the Stow-Severinghaus electrode for PtcCO_2_ measurement in a single sensor, which is also equipped with a heating element to increase the local perfusion Figure 1(a). The sensor is attached to the ear by a clip having an adhesive holder and a reflective element placed onto the inner surface.

Like other similar devices, the TOSCA pulse oximeter determines SaO_2_ by measuring the difference in absorption of selected light wavelengths by oxyhemoglobin and deoxyhemoglobin. It utilizes two light-emitting diodes (LEDs): one LED emits red light (LED-R; 658 nm wavelength) selectively adsorbed by deoxyhemoglobin, while the other one emits infrared light (LED-IR; 880 nm wavelength) selectively absorbed by oxyhemoglobin. The sensor also contains a photodiode (photodetector), an electronic device which converts light into an electronic signal proportional to the incident light intensity and which sends it to the TOSCA monitor for calculation. Unlike other pulse oximeters, either the LEDs or the photodiode are positioned on the same surface, since the light emitted is reflected by the clip surface opposite to the sensor place. The TOSCA pulse oximeter is equipped with Masimo SET technology and uses a set of algorithms designed to improve SpO_2_ monitoring during motion and low perfusion.

Transcutaneous measurement of CO_2_ is based on the observation that this gas has a high tissue solubility and diffusion through the skin, and that the application of local heat dilates blood vessels and enhances skin permeability. The PCO_2_ component of the TOSCA consists of a Stow-Severinghaus type electrode, a potentiometric sensor combining a silver/silver chloride reference electrode, and a glass pH electrode. Over the sensor surface there is an electrolyte solution, provided within a thin hydrophilic spacer and coupled to the skin via a highly gas permeable hydrophobic membrane. PCO_2_ determines a pH change of this electrolyte solution by diffusing through the skin to the sensor. The equation below illustrates the reaction occurring between carbon dioxide and water:

(1) $${\text{CO}}_{2}+{\text{H}}_{2}\text{O}↔{\text{H}}_{2}{\text{CO}}_{3}↔{\text{H}}^{+}+{\text{HCO}}^{3-}$$

Generation of hydrogen ions at the glass electrode is directly proportional to the amount of carbon dioxide which is present. An increase in the hydrogen ion concentration leads to a fall in pH and this change is proportional to the logarithm of the PCO_2_ change.

The heating element, increasing local perfusion, induces the arterialization of the capillary blood flow. However, due to the elevated temperature of the sensor, the transcutaneous PCO_2_ results higher than the arterial value mostly due to two factors [2]: first, the elevated temperature increases local blood and tissue PCO_2_ (“*anaerobic factor*”); second, the epidermal cells produce CO_2_, which contributes to the capillary CO_2_ concentration by a constant amount (“*metabolic constant*”). As a consequence, in order to provide a monitor readout corresponding as close as possible to PaCO_2_, the device applies an algorithm correction of the transcutaneous values as already specifically described by Severinghaus [3].

Since the monitor automatically performs calibration, the sensor is steadily ready to use. The small-sized sensor is easily attached to the ear lobe with a low pressure clip, and has been tested in adults [4–6], children [7], and neonates with birth weight (BW) >1,500 g and gestational age (GA) >28 weeks [8,9]. TOSCA monitor resulted reliable and applicable also in very low birth weight infants when the sensor was placed at the ear pinna, rather than at the earlobe, because of its small size. Figure 1(b) [10].

Since the use of pulse oximetry immediately after birth is indicated to titrate oxygen supplementation, as recommended by the Guidelines for Neonatal Resuscitation [11], the present study was designed to test the usability and reliability of TOSCA in the delivery room (DR) among term, late-preterm, and preterm neonates.

## 2. Experimental Section

### 2.1. Materials and Methods

This prospective observational study was carried out between January and October 2010. It was approved by the ethical committee of our institution and oral informed parental consent was obtained. The study was conducted throughout three different periods among neonates recruited immediately after birth.

During the first period (***period A***), usability and reliability of TOSCA (TOSCA^500®^ Radiometer, Copenhagen, Denmark) were evaluated in 30 term healthy neonates. A simultaneous SpO_2_ monitoring with TOSCA and pulse oximeter MASIMO Radical-7 SET (MASIMO^®^ Corporation, Irvine, CA, USA) was performed. SpO_2_ values reported by TOSCA (SpO_2TOSCA_) were compared to those reported by MASIMO (SpO_2MASIMO_) throughout 10 min of monitoring. During this period, also PtcCO_2_ levels were recorded with TOSCA (PtcCO_2TOSCA_) and a blood gas analysis (BGA) by capillary sampling was performed at the end of monitoring.

During the second period (***period B***), PtcCO_2TOSCA_ levels were recorded in 10 late-preterm neonates and compared to PtcCO_2_ values (PtcCO_2TINA_) recorded by a transcutaneous device (TINA^®^ TCM3, Radiometer, Copenhagen, Denmark). TINA was equipped with a sensor applied on the abdomen and set at 44 °C. After 10 minutes of monitoring, a BGA was performed from umbilical line.

In the third period (***period C***), 15 preterm neonates were monitored. SpO_2TOSCA_ and SpO_2MASIMO_ values were recorded immediately after birth and during stabilization and transport to the neonatal intensive care unit (NICU) (total time: 40 min). Also PtcCO_2TOSCA_ values were registered during the same period and a BGA was performed after 10 min of monitoring from umbilical line.

TOSCA was always used in QUICKSTART mode (temperature set at 44 °C during the first 20 min, then at 42 °C). The sensor probe cleaned with alcohol and dried before each application was applied to the adhesive clip at the ear pinna, using one drop of contact solution. Since Lacerenza *et al.*, showed that the placement site at the ear pinna facilitated the application of the sensor, it was electively applied at the right ear pinna rather than at the ear lobe [10]. The MASIMO sensor was placed around the right hand and applied before connection to the oximeter [12,13].

Blood samples were analyzed for PCO_2BGA_ with a blood gas analyzer (Stat Profile® Critical Care Xpress, Nova Biomedical Corporation, Waltham, MA, USA), routinely calibrated according to the manufacturer's instructions. BGA was performed at 10 min of monitoring since this time is indicated as an adequate time for TOSCA equilibration by the manifacturers themselves [14,15].

Patient characteristics (GA, BW, gender) and resuscitative measures required at birth were recorded.

The conduct of the study did not interfere with routine clinical practice: while a neonatologist was assisting the neonate, a nurse placed the pulse oximeter sensor, and a dedicated researcher applied both TOSCA and TINA sensors and collected data. Transcutaneous monitoring began as the sensors were adequately placed. Data were collected every min during the first 5 min, then every 5 min for the remaining time. At the end of the monitoring phase, sensors were removed and the underlying skin was examined to verify the presence of burns.

### 2.2. Statistical Analysis

Statistical analysis was performed using the Stata Statistical Software: Release 10 (StataCorp LP, College Station, TX, USA). Results are presented as mean ± standard deviation (SD) for continuous variables or as median (interquartile range) and as number (percentage) for categorical variables. Unpaired Student's *t* test was used for parametric data, Wilcoxon rank-sum test (Mann-Whitney U test) for nonparametric data, and Fisher's exact test for categorical variables. Bland-Altman analysis [16] was used to evaluate the agreement at 10 min between PtcCO_2TOSCA_ and PCO_2TINA_, PtcCO_2TOSCA_ and PCO_2BGA_, PCO_2BGA_ and PtcCO_2TINA_. Precision was defined as 2 SD of the mean difference (bias). A *p* < *0.05* was considered statistically significant.

## 3. Results

**Figure 2.**
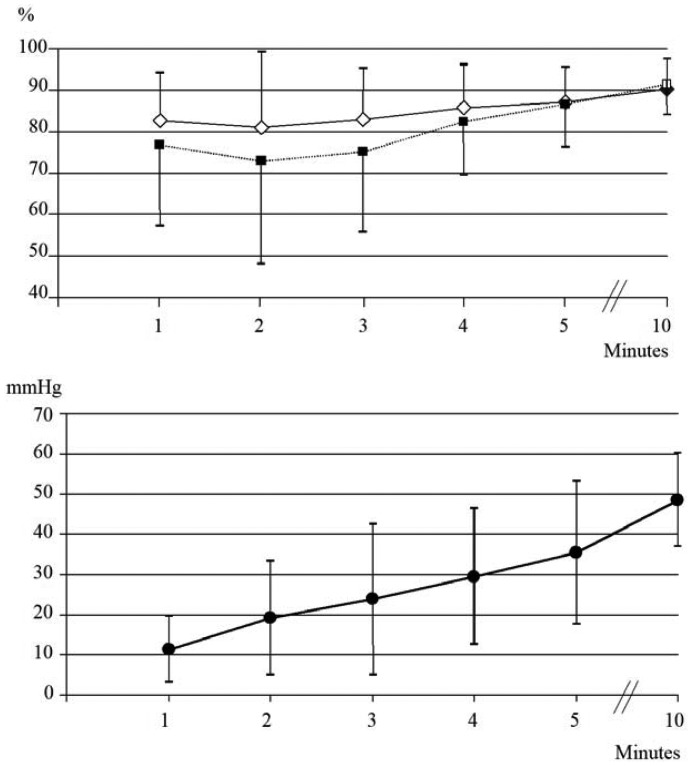
Trend of SpO_2MASIMO_ (white diamonds) and SpO_2TOSCA_ (black squares) (**top**), and trend of PtcCO_2TOSCA_; (**bottom**) during period A.

Of the 30 neonates born during ***period A*** (GA: 37.9 ± 1 weeks; BW: 3138 ± 459 g, 13 males and 17 females), none needed respiratory support at birth. No differences between SpO_2TOSCA_ and SpO_2MASIMO_ were detected at all time points (Figure 2(top)). For both monitors, data were available 60 s after sensor placement. The clipping of the TOSCA sensor was easy. Sensor repositioning was necessary in 16/30 registrations (53%) with TOSCA and in 5/30 registrations (16.6%) with MASIMO (*p* = *0.003*). After sensor repositioning, time required to obtain a stable reading was shorter for TOSCA than for MASIMO (about 10 *vs.* 15 s). PtcCO_2TOSCA_ progressively increased during the whole monitoring (Figure 2(bottom)).

PCO_2BGA_ at 10 min was 61.5 ± 10.8 mmHg, significantly higher than PtcCO_2TOSCA_ (48.6 ± 11.7 mmHg; *p* < *0.005*). At 10 min, the bias (precision) between PtcCO_2TOSCA_ and PCO_2BGA_ values was 12.9 (26.4). No signs of skin erythema or burn at the ear pinna were found at the end of monitoring.

**Figure 3.**
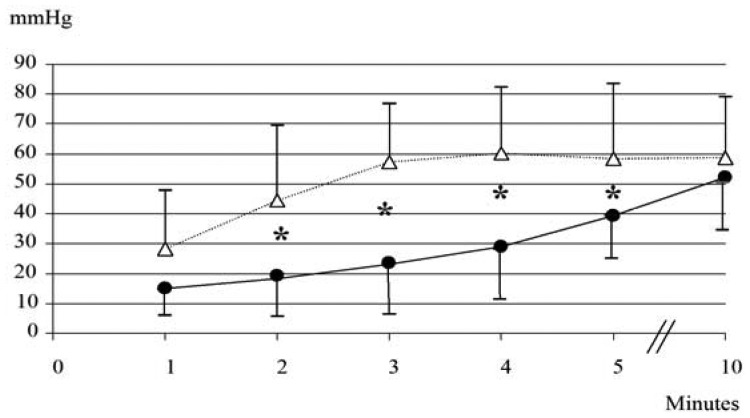
Trend of PtcCO_2TOSCA_ (black dots) and PtcCO_2 TINA_ (white triangles) during ***period B***. * *p* < 0.05.

None of the 10 neonates (GA: 36.1 ± 1.4 weeks; BW: 2743 ± 714 g; five males and five females) born during ***period B*** needed respiratory support. As shown in Figure 3, during the first 5 min PtcCO_2TOSCA_ values resulted significantly lower than PtcCO_2TINA_ values, while at 10 min similar values were recorded (PtcCO_2TOSCA_ 52 ± 17.6 mmHg *vs.* PtcCO_2TINA_ 58.6 ± 20.2 mmHg; *p* = *ns*).

PCO_2BGA_ at 10 min was 59.4 ± 17.3 mmHg; the bias (precision) between PtcCO_2TOSCA_ and PCO_2BGA_ values was 7.42 (39.8), between PCO_2BGA_ and PtcCO_2TINA_ 0.82 (31.9), and between PtcCO_2TOSCA_ and PtcCO_2TINA_ 6.6 (43.4). Sensor repositioning was required 3 times (30%) for TOSCA.

**Figure 4.**
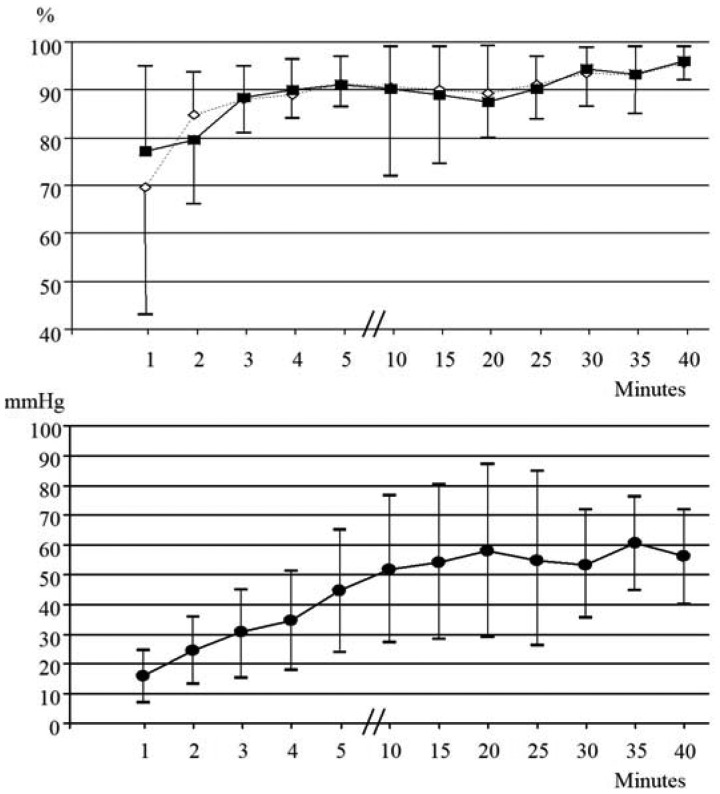
Trend of SpO_2MASIMO_ (white diamonds) and SpO_2TOSCA_ (black squares) (**top**), and trend of PtcCO_2TOSCA_ (**bottom**) during period C.

Fifteen preterm neonates (GA: 31.6 ± 2.0 weeks; BW: 1622 ± 569 grams; nine males and six females) born during ***period C*** were monitored for 40 min with TOSCA and MASIMO. Two neonates required resuscitation and were transferred to NICU mechanically ventilated; six neonates were assisted with nasal continuous positive airway pressure. Sensor repositioning was required six times (40%) for TOSCA and never for MASIMO. SpO_2TOSCA_ and SpO_2MASIMO_ values were similar during the whole study period (Figure 4(top)). PtcCO_2TOSCA_ values progressively increased until 10 min, then became stable (Figure 4(bottom)).

PCO_2BGA_ at 10 min was 63.1 ± 20.3 mmHg and the bias (precision) between PtcCO_2TOSCA_ and PCO_2BGA_ was 11.1 (43.4).

## 4. Discussion

The Guidelines on Neonatal Resuscitation recommend the use of pulse oximetry to monitor neonates' oxygenation status in the DR [11], since skin color is only partially reliable [17]. Moreover, awareness about neonatal oxygenation status is helpful to titrate oxygen supplementation, if requested.

In case of frequent motion artifacts or low perfusion, often occurring in DR, pulse oximeters with MASIMO Signal Extraction Technology are indicated [18].

TOSCA monitor records SpO_2_ with MASIMO technology and simultaneously detects PtcCO_2_, allowing a combined assessment of both parameters. The present study *investigated for the first time the possible use of TOSCA monitor in neonates immediately after birth*, a period in which the use of excessive pressure or volume (overstretching) and oxidative aggression (reactive oxygen and nitrogen species) may cause acute damage to the lung and other organs, in particular in preterm babies. The simultaneous measurement of SpO_2_ and PtcCO_2_ could be helpful both to verify the physiologic changes occurring during transition from the intra- to the extrauterine life and to determine the appropriate grade of respiratory support when resuscitation is required.

Concerning usability, our results showed that TOSCA sensor can be easily applied, even in the DR immediately after birth, to the ear pinna of both term and preterm neonates. As previously demonstrated [10], sensor application to the ear pinna rather than to the ear lobe did not alter data displaying. The size of the clipped sensor is well suited for the thin, flat, and soft ear pinna of the newborn, while it may result too large compared to the small ear lobe. Nevertheless, dislocations occurred more often using TOSCA than MASIMO. In this study, repositioning of the TOSCA sensor was more frequently required among term than among late-preterm or preterm neonates, presumably because of their greater mobility. However, the viability of TOSCA monitoring was not impaired thanks both to the shorter time required for sensor clipping and to its faster reading, due to the high sensor's stability [1,2].

No burns underneath the TOSCA sensor were identified in any of the infants, including premature neonates monitored for longer period (40 min) in period C. This was presumably due to a lower electrode temperature after 20 min of monitoring in the QUICKSTART mode. In fact, this modality allows a long term (up to 12 h) application of the sensor without adverse events, although measurements of PtCO_2_ may result less reliable [9]. This could be due to the lower sensor temperature which is not able to provide an adequate grade of arterialization in order to compensate the greater CO_2_ local production induced by temperature itself. However, this occurrence has only limited implications in our study, since we performed a “long time evaluation” of the PtcCO_2TOSCA_ exclusively in Period C, throughout a monitoring period which is much shorter than the one described by other authors [9].

Concerning the reliability of SpO_2TOSCA_, the recorded values were comparable to those detected by SpO_2MASIMO_. As formerly demonstrated [10], the different placement site of both sensors, always preductal, did not affect SpO_2_ detection.

Regarding PtcCO_2TOSCA_ measurement in DR, the estimation accuracy of TOSCA resulted generally acceptable compared to blood samples collected at 10 min of use. Although this single BGA, representing a punctual evaluation, may be a limit in the estimation of PtcCO_2_ values, a higher number of blood collection was avoided to prevent painful punctures. However, the agreement between PtcCO_2TOSCA_ and PCO_2BGA_ was more accentuated when blood samples were taken by the umbilical line (period B and C) compared to the capillary sampling (period A). A possible explanation of these results may be that capillary PCO_2BGA_ is comparable to arterial PCO_2BGA_ only after the first hours of life [19]. Nevertheless, after 10 min of registration, the TOSCA monitor reveals less accurate PtcCO_2_ values than the TINA one compared to the umbilical PCO_2BGA_ (period B), confirming that the measurement of PtCO_2_ with TOSCA was less precise than the conventional monitoring [10].

The results achieved throughout ***period B*** demonstrated that during the first minutes of registration the PtcCO_2TOSCA_ values were lower than those recorded by TINA and became comparable only after 5 min. This is in agreement with previously reported data achieved among preterm neonates admitted to NICU, and was probably dependent on the placement site of the TOSCA sensor [8]. The thin dermal layer and the poor capillary bed of the ear pinna may induce a lower grade of arterialization than the one obtained in other sites [10].

A similar slowly increasing trend of the PtcCO_2TOSCA_ was also detected among preterm infants monitored after birth and during transport to the NICU (period C), and was followed by value stabilization after 10 min of monitoring.

## 5. Conclusions

We can assert that TOSCA was easily and safely usable in DR both in term and preterm neonates, allowing a reliable and noninvasive estimation of SpO_2_. Considering that at least 10 min were required to obtain reliable and stable values of PtcCO_2TOSCA_, TOSCA reliability results limited during the first minutes of life, when resuscitation procedures may be necessary. However, even if the optimal level of PCO_2_ during neonatal resuscitation is not known yet [20], PtcCO_2_ measurement with TOSCA could be useful as trend monitoring after neonatal stabilization and during transport to the NICU to prevent lung injury, since its beginning may occur soon after birth among neonates requiring positive-pressure ventilation. We believe that our study may represent a stimulating starting point for further investigations focusing on the development of respiratory function monitoring devices to be used in DR [21]. In the near future, an efficient technology may enable the individualization of the specific ventilatory and oxygenation requirements, thus minimizing physical and oxygen-derived damage [22].
